# Clinical outcomes of adding vincristine to procarbazine-lomustine in patients with gliomas: insights from current evidence

**DOI:** 10.3332/ecancer.2026.2115

**Published:** 2026-05-01

**Authors:** Viviana Pinzón-Ramírez, Luis E Cueva-Cañola, Dilmareth E Natera, Helder Edgar Aldo-Chávez Olivera, Oscar Eduardo Camacho-Hernández, Andrea C Beltran-De la Fuente, Sergio Alexis Ramirez-Alvarez, Einstein Yhair Gallardo Cubas, Giomar Vilca Flores, Mauricio E Gamez, Leonardo Rangel Castilla

**Affiliations:** 1 School of Medicine, Universidad Pedagógica y Tecnológica de Colombia (UPTC), Tunja, Boyacá 150003, Colombia; 2 Neurology Research Club, Universidad Nacional de Piura (UNP), Piura 20000, Peru; 3 Department of Neurosurgery, Instituto Nacional de Ciencias Neurológicas (INCN), Lima 15003, Peru; 4 Department of Neurosurgery, Hospital III-1 EsSalud José Cayetano Heredia, Piura 20007, Peru; 5 Department of Neurosurgery, University of Minnesota, Minneapolis, MN 55455, USA; 6 Department of Medicine and Nutrition, Health Sciences Division, University of Guanajuato, Leon Campus, Leon, Guanajuato 37544, Mexico; 7 School of Medicine, Universidad México Americana Del Norte (UMAN), Reynosa, Tamaulipas 88500, México; 8 School of Medicine, Universidad Autonoma de Guadalajara (UAG), Guadalajara, Jalisco 45129, Mexico; 9 School of Medicine, Universidad Católica de Santa María, Umacollo, Arequipa 04017, Peru; 10 Creo Cancer Center, Universidad Autonoma de San Luis Potosi (UASLP), San Luis Potosi 78210, Mexico; 11 Department of Neurosurgery, Hospital Lomas International, San Luis Potosí 78218, Mexico; a https://orcid.org/0009-0008-6525-6468; b https://orcid.org/0009-0003-6320-5767; c https://orcid.org/0009-0001-5256-9798; d https://orcid.org/0009-0000-2294-5038; e https://orcid.org/0009-0000-3141-3041; f https://orcid.org/0009-0009-9895-0824; g https://orcid.org/0009-0009-0024-288X; h https://orcid.org/0009-0000-3785-6249; i https://orcid.org/0000-0001-8232-0806; j https://orcid.org/0009-0009-0277-5698; k https://orcid.org/0000-0002-7915-6205

**Keywords:** glioma, procarbazine, lomustine, vincristine, meta-analysis

## Abstract

**Background:**

Temozolomide (TMZ) is the standard chemotherapy for gliomas due to good tolerability. Clinical studies suggest a combination of procarbazine, lomustine (CCNU) and vincristine (PCV) provides superior survival and progression delay, but toxicities limit use. Given the ongoing debate over the role of vincristine in glioma chemotherapy amid the dominance of TMZ, simplified procarbazine and CCNU (PC) regimens warrant renewed evaluation.

**Methods:**

Three databases were searched up to August 2025 to identify studies that directly compared PC and PCV in patients with low-grade and high-grade gliomas. Outcomes included progression-free survival (PFS), overall survival (OS) and treatment-related toxicities. Hazard ratios (HRS) and relative risks (RRS) were then pooled using a random-effects model.

**Results:**

In a total population of 301 patients with low- and high-grade gliomas, PC significantly reduced the risk of disease progression compared with PCV (HR = 0.72, 95% confidence interval (CI): 0.53–0.98; *p* = 0.04) and OS was also significantly longer with PC (HR = 0.59, 95% CI: 0.37–0.95; *p* = 0.03). Neurotoxicity (RR = 0.12, 95% CI: 0.02–0.63; *p* = 0.01) and treatment discontinuation (RR = 0.11, 95% CI: 0.02–0.80; *p* = 0.03) were less frequent with PC.

**Conclusion:**

With the limited evidence available to date, we found that PC achieved significantly longer PFS and OS than PCV, while maintaining a better safety profile. We recommend further well-designed and adequately powered randomised clinical trials directly comparing TMZ, PC and PCV to determine which patient populations and glioma subtypes – according to the current World Health Organisation 2021 classification – derive the greatest survival benefit and tolerability from each regimen.

**Key points:**

- Procarbazine–lomustine combination (PC) shows superior progression-free survival and overall survival versus procarbazine, lomustine and vincristine (PCV) in gliomas.
- Omitting vincristine may improve efficacy and reduce neurotoxicity.
- Findings suggest PC could be a safer alternative, pending confirmation by randomised controlled trials.

**Importance of the study:**

This meta-analysis is the first to directly evaluate whether vincristine is necessary within the procarbazine, lomustine and vincristine (PCV) regimen for gliomas. By synthesising data from three retrospective cohorts, it demonstrates that the procarbazine–lomustine combination (PC) achieved significantly longer progression-free and overall survival compared with PCV, while markedly reducing neurotoxicity and treatment discontinuation. These findings challenge the traditional view that vincristine is an indispensable component of chemotherapy for diffuse gliomas. Given that temozolomide has become the preferred agent in contemporary practice, clarifying the relative contributions of PC, PCV and TMZ remains essential for optimising therapeutic strategies. Although limited by the small number and retrospective design of included studies, this analysis suggests that PC may represent a more effective and tolerable alternative, underscoring the need for prospective randomised trials in the modern molecular era of glioma classification.

## Introduction

Diffuse gliomas account for 77%–80% of malignant primary brain tumours and nearly 30% of all primary brain tumours worldwide. These include both low-grade (World Health Organisation (WHO) grade II) and high-grade (WHO grade III–IV) gliomas [1]. Since the 1990s, adjuvant chemotherapy has become an essential component of glioma management [2, 3]. The combination of procarbazine, lomustine (CCNU) and vincristine (PCV), when added to radiotherapy, has demonstrated significant improvements in overall survival (OS) and progression-free survival (PFS) in randomised controlled trials (RCTs), particularly among patients with anaplastic oligodendrogliomas harboring 1p/19q–codeleted, in whom PCV plus radiotherapy outperformed radiotherapy alone [4, 5].

In current practice, however, temozolomide (TMZ) has largely replaced PCV because of its oral administration, favourable toxicity profile and improved tolerability [6]. Nonetheless, evidence indicates that PCV offers superior efficacy compared to TMZ, especially in WHO grade III isocitrate dehydrogenase (IDH)-mutant oligodendroglioma 1p/19q–codeleted [7, 8]. The major limitation of PCV is its toxicity: grade 3–4 hematologic toxicities, reported in up to 40% of patients, often necessitating early treatment discontinuation [9–11].

Within the PCV regimen, the therapeutic role of vincristine has increasingly been questioned for several reasons, including concerns related to its tolerability, pharmacological properties and overall contribution to treatment efficacy [12]. Emerging data suggest that omitting vincristine, using procarbazine and CCNU (PC) alone, may maintain the survival benefit while reducing toxicity [13].

Comparative data evaluating modified regimens, such as PC without vincristine or TMZ-based approaches, remain limited and largely retrospective [6, 14]. Previous systematic reviews have focused broadly on chemotherapy in gliomas but have not specifically addressed the necessity of vincristine in oligodendroglioma treatment [6]. Given the long survival trajectories of these patients, clarifying the role of vincristine is crucial for balancing efficacy with toxicity.

The objective of this meta-analysis is to synthesize the available evidence from three retrospective cohort studies comparing procarbazine–CCNU regimens with and without vincristine in patients with low- and high-grade gliomas.

## Methods

This systematic review and meta-analysis were conducted in accordance with the guidelines of the Preferred Reporting Items for Systematic Reviews and Meta-Analyses (PRISMA), which establish a structured approach to enhance clarity and reliability in reporting [15]. In addition, to ensure methodological transparency and minimise the possibility of duplication or selective outcome reporting, the protocol was prospectively registered in the International Prospective Register of Systematic Reviews on 25 August 2025 (registration ID: CRD420251134032) [16].

### Eligibility criteria

We included studies that enrolled patients with histologically confirmed low- or high-grade gliomas, directly compared PC with PCV and reported at least one relevant clinical outcome. After a comprehensive screening, only three retrospective cohort studies meeting these criteria were identified and included in the synthesis. Studies were excluded if they were preclinical reports, reviews, meta-analyses, case reports, case series, lacked a direct PC versus PCV comparison or were published in languages other than English.

### Search strategy

We systematically searched PubMed, Embase and Web of Science for studies published up to 13 August 2025. The search strategy combined controlled vocabulary and free-text terms related to glioma, procarbazine, lomustine and vincristine. The detailed search strategies for each database are provided in Table S1.

### Screening of studies

All records retrieved from the database search were imported into Zotero for reference management, where duplicates were removed. The remaining studies were screened in Rayyan through a two-step process. In the first stage, two independent reviewers (SRA and HCO), who were blinded to each other’s decisions, screened titles and abstracts to identify potentially eligible studies. In the second stage, the same reviewers independently assessed the full texts for final inclusion. Any disagreements were resolved through discussion, and if consensus could not be achieved, a third reviewer served as arbitrator (LCC).

### Data extraction

Two reviewers (SRA and HCO) independently extracted data using a standardised form, with disagreements resolved by a third reviewer (LCC). Extracted information included study characteristics, treatment details, baseline patient and tumour features and reported clinical outcomes. When necessary, corresponding authors were contacted for clarification and all data were cross-checked for accuracy before analysis.

### Outcomes

The predefined outcomes of interest were categorised into efficacy and safety endpoints. Efficacy was evaluated through PFS and OS, which represent the length of time patients lived without disease progression and the survival time from treatment initiation, respectively [6]. Safety outcomes included the occurrence of adverse events, as well as treatment modifications, dose reductions and dose delays, which reflect treatment tolerability in clinical practice [14]. These endpoints were consistently extracted from the included studies, using the longest follow-up available when multiple time points were reported.

### Statistical analysis

For time-to-event outcomes (PFS and OS), hazard ratios (HR) with 95% confidence intervals (CIs) were either extracted directly or calculated from available data. The pooled analyses were performed using adjusted HR. For OS, HR were adjusted for age, tumour type, IDH mutation status, 1p/19q–codeleted status, O⁶-methylguanine-DNA methyltransferase (MGMT) promoter methylation status, prior history of chemotherapy and Karnofsky performance status (KPS) [10, 11, 13]. For PFS, HR was adjusted for age, extent of resection before chemotherapy, tumour type and KPS [10, 11, 13].

For safety outcomes, relative risks (RR) with 95% CIs were used to compare adverse events, dose reductions and dose delays between treatment groups. All statistical analyses were performed using Review Manager version 5.3.5 [17].

A random-effects model was applied to account for between-study variability and pooled results were presented in forest plots. Heterogeneity was assessed using the Chi-squared test and quantified with the I² statistic, with I² ≥ 50% or p < 0.10 were considered indicative of substantial heterogeneity [18].

Given that only three retrospective cohort studies met the inclusion criteria, the scope of the statistical analysis was limited. With such a small number of studies, advanced approaches such as meta-regression, subgroup analyses or formal assessments of publication bias (funnel plots and Egger’s test) were not feasible, as they would provide unreliable or uninterpretable results [19].

### Risk-of-bias assessment

The risk of bias was assessed using the Risk Of Bias In Non-randomised Studies of Interventions tool, which covers confounding factors, participant selection, classification of interventions, deviations from planned interventions, missing data, outcome measurement and selective reporting [20]. Two reviewers performed the assessment blindly and a third reviewer resolved any disagreements.

## Results

### Study selection

**Figure 1.**
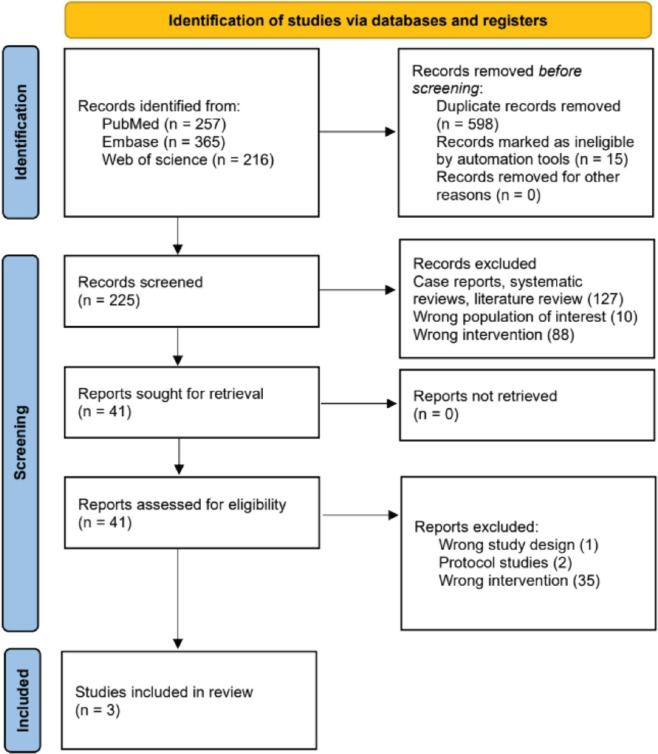
PRISMA 2020 flow diagram.

A total of 838 records were identified through the database search. After removal of duplicates and screening of titles and abstracts, 41 full-text articles were reviewed in detail to evaluate their eligibility. Ultimately, only three studies met all eligibility criteria and were included in the final synthesis. All three were retrospective cohort studies conducted in Germany [10], South Korea [13] and the United States [11]. The study selection process is depicted in the PRISMA flow diagram (Figure 1).

### Included population

In our study, we analysed 301 patients (187 men and 114 women) with a mean age of 44.2 ± 12.3 years. Of these, 88 (29.2%) had low-grade and 213 (70.8%) high-grade gliomas. Regarding surgical management, 190 (63.1%) patients underwent maximal safe resection at initial surgery. Baseline clinical and molecular characteristics are shown in Table 1, while detailed treatment regimens are summarised in Table S2.

### Efficacy of chemotherapy regimens

**Figure 2.**
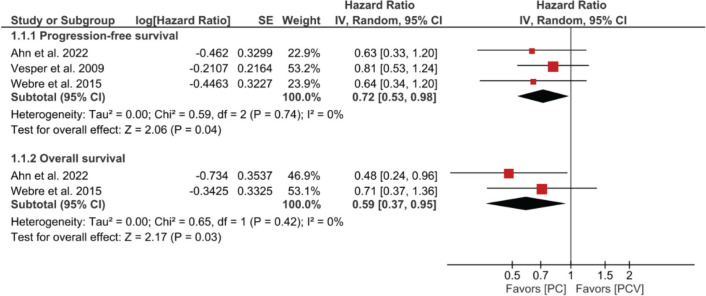
Efficacy of treatment regimens HR analysis.

Patients receiving PC had a significantly lower risk of disease progression compared with those receiving PCV (HR = 0.72, 95% CI: 0.53–0.98, *p* = 0.04). Similarly, OS was significantly longer in the PC group than in the PCV group (HR = 0.59, 95% CI: 0.37–0.95, *p* = 0.03). No heterogeneity was detected across studies for either outcome. These analyses are shown in Figure 2.

### Treatment-related toxicities and tolerability

**Figure 3.**
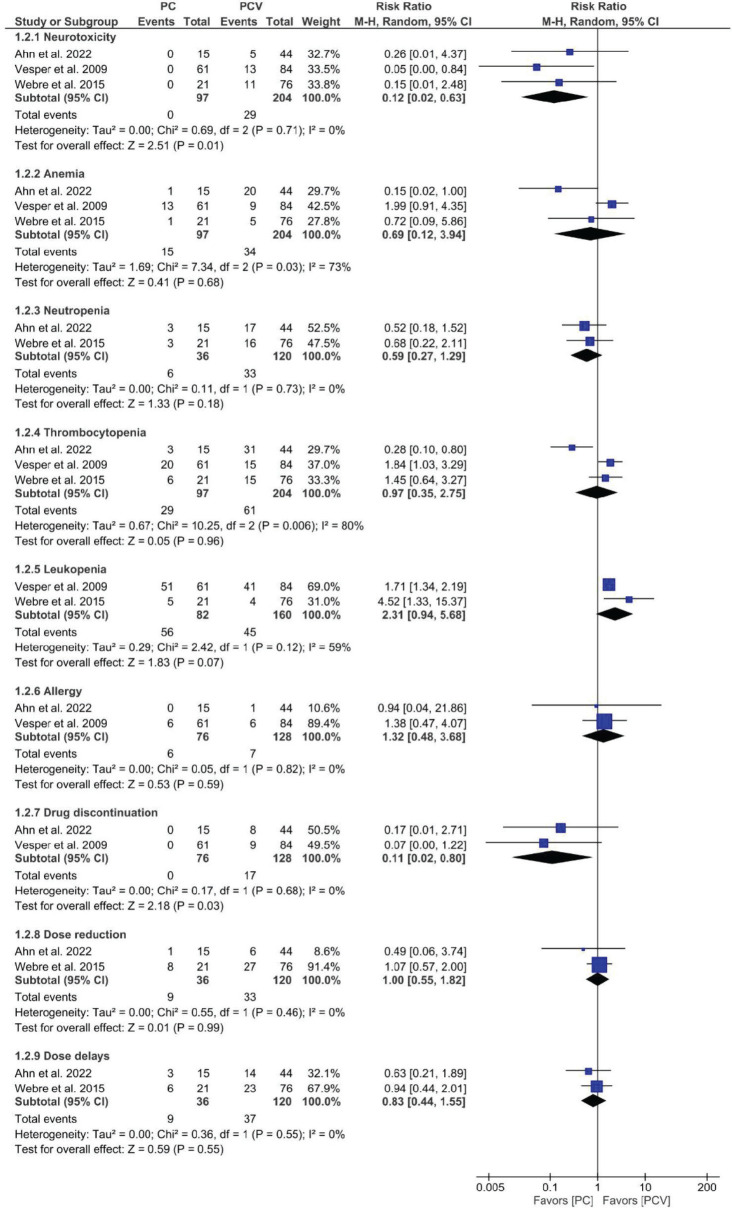
Tolerability of treatment regimens RR analysis.

The pooled analyses revealed that neurotoxicity was significantly less frequent in patients treated with PC compared with PCV (RR = 0.12, 95% CI: 0.02–0.63, *p* = 0.01; *I*² = 0%). Similarly, treatment discontinuation due to adverse events was markedly lower with PC (RR = 0.11, 95% CI: 0.02–0.80, *p* = 0.03; *I*² = 0%). These analyses are shown in Figure 3.

For hematologic toxicities, no statistically significant differences were observed between groups. Anemia, neutropenia, thrombocytopenia and leukopenia occurred at comparable rates. Likewise, no significant differences were found in allergic reactions, dose reductions or dose delays.

### Risk-of-bias assessment

**Figure 4.**
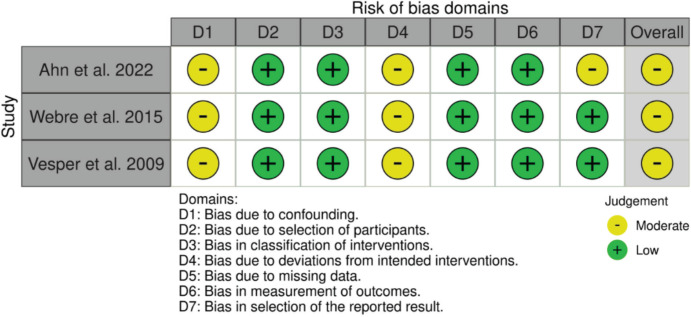
Risk of bias assessment.

All studies were judged to have a moderate overall risk of bias (Figure 4). The main sources of bias across the included studies were related to their retrospective, non-randomised design, which makes residual confounding likely and the absence of pre-registered protocols, raising the possibility of selective reporting. Additional concerns included treatment deviations in the PCV groups due to toxicity and the inherent limitations of retrospective case capture. However, because important clinical and molecular variables were reported and adjusted for, patient selection was generally appropriate. The key outcomes (OS, PFS, toxicity) were consistently presented, so the overall risk of bias was judged to be moderate rather than serious [10, 11, 13].

## Discussion

In contemporary clinical practice, TMZ has become the most commonly prescribed chemotherapy for IDH-mutant diffuse gliomas classified according to the WHO grading system, because of its more favorable tolerability profile [21]. However, several studies directly comparing TMZ with PCV have demonstrated that PCV, when combined with radiotherapy, achieves superior outcomes in terms of OS and PFS, albeit at the cost of greater treatment-related toxicity [7, 8, 22–25]. This trade-off between efficacy and tolerability has generated ongoing debate regarding the optimal chemotherapy backbone in this patient population.

To the best of our knowledge, this is the first meta-analysis to provide quantitative evidence that omitting vincristine from the PCV regimen not only preserves but may enhance therapeutic efficacy while substantially improving tolerability. Patients receiving PC had a 41% lower risk of death and a 28% lower risk of disease progression compared with those treated with PCV. These findings suggest that the inclusion of vincristine may not only fail to improve outcomes but could, under certain conditions, be counterproductive. While vincristine has long been considered an integral component of the PCV regimen, its actual contribution to intracranial tumour control has remained uncertain.

Procarbazine exerts its cytotoxicity primarily through the generation of O⁶-methylguanine (O⁶-MeG) adducts, lesions whose biological impact depends critically on deoxyribonucleic acid (DNA) replication and intact mismatch repair (MMR) [26]. During the first Synthesis phase (S phase) after exposure, O⁶-MeG mispairs with thymine, prompting futile MMR cycles that persist into the second cell cycle, when long single-stranded DNA stretches and double-strand breaks emerge and activate Ataxia Telangiectasia and Rad3-related/Ataxia Telangiectasia Mutated, Checkpoint Kinase 1/ Checkpoint Kinase 2, p53 and downstream apoptosis or senescence pathways [21, 27]. CCNU also relies on replication-associated lesion processing: its O⁶-chloroethylguanine adducts convert into interstrand crosslinks (ICLs), whose collapse during replication fork collision triggers double-strand break (DSB) formation, checkpoint activation and cell death [27]. Thus, although ICLs possess intrinsic cytotoxicity, their full lethal potential is amplified when cells progress through S phase, a feature shared with O⁶-alkylating agents.

Vincristine disrupts this replication-dependent cytotoxic cascade by binding β-tubulin, inhibiting microtubule polymerisation and inducing a durable Gap 2 / Mitosis phase arrest [28]. This mitotic blockade markedly reduces the proportion of cells entering successive S phases – the very intervals during which O⁶-MeG lesions and chloroethyl adducts undergo the replication-dependent processing that converts them into DSBs and ICL-derived fork collapse [21, 29, 30].

Additionally, it is well known that vincristine exhibits low penetration across an intact blood–brain barrier, and although regions of tumour-associated barrier disruption may permit somewhat higher local exposure, its cytotoxic activity remains strictly dependent on active cell division [12, 31, 32].

The omission of vincristine would not only avoid the neurotoxicity related to its binding to neuronal β-tubulin, which presents as impaired axonal transport, peripheral neuropathy and autonomic dysfunction, but would also prevent the dose reductions and treatment interruptions that these toxicities often require [11, 33–35]. Removing this limiting factor may help support more consistent full-dose administration of procarbazine and CCNU, which in turn could facilitate the pharmacodynamic interactions they are expected to rely on through sustained exposure, progressive MGMT depletion and the replication-linked conversion of monoadducts into cytotoxic DNA lesions.

The absence of RCTs directly comparing PC and PCV regimens can be attributed to several practical and ethical barriers. Diffuse gliomas are relatively rare tumours, and demonstrating meaningful differences in OS or PFS would require very large patient cohorts and extended follow-up periods, making such trials logistically and financially challenging [36, 37]. Moreover, once PCV demonstrated survival benefit in earlier studies, it became established as part of the standard of care, reducing clinical equipoise and making it ethically difficult to randomise patients to modified regimens that omit vincristine [7, 8, 25]. Therefore, most available evidence comes from retrospective or observational studies, which are inherently vulnerable to selection bias and confounding by patient characteristics.

The therapeutic landscape of diffuse gliomas is increasingly shaped by molecular profiles, with treatment strategies varying according to grade and risk. For high-risk WHO grade 2 tumours, radiotherapy followed by PCV chemotherapy remains the standard of care, supported by level IA evidence [38], while the recent Food and Drug Administration approval of vorasidenib for IDH1/2-mutant gliomas underscores the role of targeted therapies [39]. In higher grades, management continues to rely on established regimens such as the Stupp protocol for glioblastoma and radiotherapy combined with either TMZ or PCV for anaplastic astrocytomas, particularly in IDH-mutant and 1p/19q–codeleted tumours [40]. Long-term data have shown that PCV provides a significant survival advantage and delays progression in IDH-mutant grade 3 diffuse gliomas compared with TMZ [7, 8]. Our results, however, suggest that this benefit may be driven primarily by the alkylating components of PCV, and that removal of vincristine not only preserves but enhances efficacy while substantially reducing toxicity.

While this study presents interesting findings, several limitations should be acknowledged. The small number of available studies, their retrospective design and variability in treatment details limit the strength of our conclusions; therefore, these results should be interpreted with caution. Our study population was broad and included Ahn *et al* [13], which evaluated recurrent gliomas, Webre *et al* [11], focused on anaplastic oligodendroglial brain tumours and Vesper *et al* [10], which included both low- and high-grade oligodendroglial tumours. Notably, these studies followed different WHO classifications – 2000 for Webre, 2000 for Vesper and 2016 for Ahn – further limiting comparability of results. In addition, the limited dataset prevented meaningful subgroup analyses.

Clinicians should interpret these findings with caution, given the absence of RCTs and the inherent limitations of retrospective data. However, the consistency of the observed survival benefit, together with the substantially improved tolerability profile and the strong mechanistic rationale supporting vincristine omission, suggests that the PC regimen represents a clinically meaningful and biologically plausible alternative to PCV.

Rather than prompting immediate changes to universal treatment guidelines, our results support the consideration of vincristine-free regimens in carefully selected clinical contexts, particularly for patients at increased risk of neurotoxicity or those unable to tolerate full-dose PCV. In this sense, the present meta-analysis provides a robust evidence-based framework that may assist clinicians and institutions in rationally evaluating and, where appropriate, implementing the PC protocol while awaiting confirmation from prospective studies. Future investigations should directly compare TMZ, PCV and PC across molecularly defined glioma subtypes, according to the WHO 2021 classification, to determine which patient populations derive the greatest survival benefit and best tolerability. These trials should also apply standardised chemotherapy dosing schedules, radiotherapy protocols and toxicity grading systems, while integrating patient-reported outcomes, neurocognitive assessments and long-term follow-up to capture delayed toxicities and survival benefit. Incorporation of translational endpoints evaluating cell cycle dynamics and DNA damage response may further clarify the biological impact of vincristine in alkylator-based regimens.

Collaborative, multicenter randomised designs and international registries will be essential to overcome the limited incidence and slow progression of these tumours. The generation of such high-quality prospective evidence will be critical to confirm the therapeutic contribution of vincristine and to optimise treatment strategies in the molecular era of glioma therapy.

## Conclusion

Our meta-analysis suggests that omitting vincristine from the PCV regimen may be associated with improved PFS and OS, while reducing neurotoxicity and treatment discontinuation. However, given that these results derive from a small number of retrospective studies with heterogeneous designs and limited molecular characterisation, they should be interpreted with caution. The observed associations do not establish causality and should be considered hypothesis-generating. Future RCTs, ideally molecularly stratified according to the WHO 2021 classification, are essential to confirm whether vincristine provides any additional therapeutic benefit within combination chemotherapy in glioma treatment.

## List of abbreviations

CCNU, lomustine; CI, confidence interval; DNA, deoxyribonucleic acid; DSB, double-strand break; HR, hazard ratio; ICL, interstrand crosslink; IDH, isocitrate dehydrogenase; KPS, Karnofsky performance status; MGMT, O⁶-Methylguanine-DNA Methyltransferase; MMR, mismatch repair; O⁶-MeG, O⁶-Methylguanine; OS, overall survival; PC, procarbazine and lomustine; PCV, procarbazine, lomustine and vincristine; PFS, progression-free survival; PRISMA, preferred reporting items for systematic reviews and meta-analyses; RCT, randomised controlled trial; RR, relative risk; S phase, synthesis phase; TMZ, temozolomide; WHO, World Health Organisation.

## Conflicts of interest

The authors declare that they have no competing interests. No financial, institutional or personal relationships influenced the design, conduct, analysis or reporting of this review.

## Funding

This study did not receive funding from any public, commercial or non-profit organisation. All stages of the research, including study design, data collection, analysis, interpretation and manuscript preparation, were conducted without external financial support.

## Ethical approval and patient consent

As this study is a systematic review and meta-analysis based solely on previously published studies, neither ethical approval nor informed consent was necessary.

## Author contributions

Viviana Pinzon-Ramirez: Conceptualisation, Methodology, Investigation, Data curation, Writing – original draft, Project administration. Luis E. Cueva-Cañola: Conceptualisation, Methodology, Investigation, Software, Validation, Writing – original draft, Formal analysis, Writing – review & editing. Dilmareth E. Natera: Investigation, Resources, Data curation, Writing – original draft. Helder Edgar Aldo-Chávez Olivera: Investigation, Resources, Data curation, Writing – original draft. Oscar Eduardo Camacho-Hernández: Investigation, Resources, Data curation, Writing – original draft. Andrea C. Beltran-De la Fuente: Investigation, Resources, Data curation, Writing – original draft. Sergio Alexis Ramirez-Alvarez: Investigation, Resources, Data curation, Writing – original draft. Einstein Yhair-Gallardo Cubas: Investigation, Resources, Data curation, Writing – original draft. Giomar Vilca Flores: Investigation, Resources, Data curation, Writing – original draft. Mauricio E. Gamez: Writing, Review & editing. Leonardo Rangel Castilla: Writing, Review & editing.

## Data transparency statement

The data extraction templates, extracted datasets and analysis codes are available from the authors upon reasonable request.

## Reporting guidelines

This review was conducted in accordance with the Preferred Reporting Items for Systematic Reviews and Meta-Analyses (PRISMA) guidelines.

## Figures and Tables

**Table 1.** Summary of included studies.

Study	Country	Study design	Tumour type	Median follow-up	Treatment	Sample	Age	Male	Biopsy only	MSR	1p/19q Codeletion	IDH mutation
Ahn *et al* [13]	South Korea	Retrospective cohort	Recurrent adult glioma (High-grade glioma)	424.6 days	PC	15	50.9 ± 17.0	11	0	15	2	3
					PCV	44	48.3 ± 11.9	27	0	44	7	13
Webre *et al* [11]	United States of America	Retrospective cohort	Anaplastic Oligodendroglial brain tumours (High-grade glioma)	9.9 years	PC	21	46.3 ± 11.1	14	1	GTR: 8 PR: 12	4	NR
					PCV	76	39.4 ± 10.4	43	8	GTR: 23 PR: 35	11	NR
Vesper *et al* [10]	Germany	Retrospective cohort	Oligodendroglial brain tumours (High- and low-grade glioma)	7 years	PC	84	45.0 ± 11.9	54	61	23	NR	NR
					PCV	61	43.8 ± 12.7	38	31	30	NR	NR

Data were expressed as mean ± standard deviation or frequency GTR: Gross Total Resection, IDH: Isocitrate Dehydrogenase, MSR: Maximal Safe Resection, NR: Not Reported, PC: Procarbazine + CCNU (Lomustine), PCV: Procarbazine + CCNU (Lomustine) + Vincristine, PR: Partial Resection

**Table S1.** Search strategy.

Database	Search strategy
Pubmed (*n* = 257)	(glioma[tiab] OR astrocytoma[tiab] OR oligodendroglioma[tiab] OR glioblastoma[tiab] OR "astrocytoma idh-mutant"[tiab] OR "glioblastoma idh-wildtype"[tiab] OR "astrocytoma pilocytic"[tiab] OR "pleomorphic xanthoastrocytoma"[tiab] OR "subependymal giant cell astrocytoma"[tiab] OR "chordoid glioma"[tiab] OR "astroblastoma mn1-altered"[tiab] OR "high-grade astrocytoma with piloid features"[tiab] OR "angiocentric glioma"[tiab] OR plnty[tiab] OR "diffuse midline glioma h3 k27-altered"[tiab] OR "diffuse hemispheric glioma h3 g34-mutant"[tiab] OR "infant-type hemispheric glioma"[tiab] OR ganglioglioma[tiab] OR "desmoplastic infantile astrocytoma"[tiab] OR "desmoplastic infantile ganglioglioma"[tiab] OR "dysembryoplastic neuroepithelial tumour"[tiab] OR dnet[tiab] OR "multinodular vacuolating neuronal tumour"[tiab] OR mvnt[tiab] OR "rosette-forming glioneuronal tumour"[tiab] OR "papillary glioneuronal tumour"[tiab] OR "diffuse leptomeningeal glioneuronal tumour"[tiab] OR "myxoid glioneuronal tumour"[tiab] OR gangliocytoma[tiab] OR "lhermitte-duclos disease"[tiab] OR "central neurocytoma"[tiab] OR "extraventricular neurocytoma"[tiab] OR "cerebellar liponeurocytoma"[tiab] OR ependymoma[tiab] OR "ependymoma zfta fusion-positive"[tiab] OR "myxopapillary ependymoma"[tiab] OR subependymoma[tiab] OR glioma[MeSH Terms]) AND ((procarbazine[tiab] AND lomustine[tiab] AND vincristine[tiab]) OR (pcv[tiab] AND pc[tiab]))
Embase (*n* = 365)	(glioma:ti,ab OR astrocytoma:ti,ab OR oligodendroglioma:ti,ab OR glioblastoma:ti,ab OR 'astrocytoma idh-mutant':ti,ab OR 'glioblastoma idh-wildtype':ti,ab OR 'astrocytoma pilocytic':ti,ab OR 'pleomorphic xanthoastrocytoma':ti,ab OR 'subependymal giant cell astrocytoma':ti,ab OR 'chordoid glioma':ti,ab OR 'astroblastoma mn1-altered':ti,ab OR 'high-grade astrocytoma with piloid features':ti,ab OR 'angiocentric glioma':ti,ab OR plnty:ti,ab OR 'diffuse midline glioma h3 k27-altered':ti,ab OR 'diffuse hemispheric glioma h3 g34-mutant':ti,ab OR 'infant-type hemispheric glioma':ti,ab OR ganglioglioma:ti,ab OR 'desmoplastic infantile astrocytoma':ti,ab OR 'desmoplastic infantile ganglioglioma':ti,ab OR 'dysembryoplastic neuroepithelial tumour':ti,ab OR dnet:ti,ab OR 'multinodular vacuolating neuronal tumour':ti,ab OR mvnt:ti,ab OR 'rosette-forming glioneuronal tumour':ti,ab OR 'papillary glioneuronal tumour':ti,ab OR 'diffuse leptomeningeal glioneuronal tumour':ti,ab OR 'myxoid glioneuronal tumour':ti,ab OR gangliocytoma:ti,ab OR 'lhermitte-duclos disease':ti,ab OR 'central neurocytoma':ti,ab OR 'extraventricular neurocytoma':ti,ab OR 'cerebellar liponeurocytoma':ti,ab OR ependymoma:ti,ab OR 'ependymoma zfta fusion-positive':ti,ab OR 'myxopapillary ependymoma':ti,ab OR subependymoma:ti,ab OR glioma/exp) AND ((procarbazine:ti,ab AND lomustine:ti,ab AND vincristine:ti,ab) OR (pcv:ti,ab AND pc:ti,ab))
Web of science (*n* = 216)	((TI=glioma OR AB=glioma) OR (TI=astrocytoma OR AB=astrocytoma) OR (TI=oligodendroglioma OR AB=oligodendroglioma) OR (TI=glioblastoma OR AB=glioblastoma) OR (TI="astrocytoma idh-mutant" OR AB="astrocytoma idh-mutant") OR (TI="glioblastoma idh-wildtype" OR AB="glioblastoma idh-wildtype") OR (TI="astrocytoma pilocytic" OR AB="astrocytoma pilocytic") OR (TI="pleomorphic xanthoastrocytoma" OR AB="pleomorphic xanthoastrocytoma") OR (TI="subependymal giant cell astrocytoma" OR AB="subependymal giant cell astrocytoma") OR (TI="chordoid glioma" OR AB="chordoid glioma") OR (TI="astroblastoma mn1-altered" OR AB="astroblastoma mn1-altered") OR (TI="high-grade astrocytoma with piloid features" OR AB="high-grade astrocytoma with piloid features") OR (TI="angiocentric glioma" OR AB="angiocentric glioma") OR (TI=plnty OR AB=plnty) OR (TI="diffuse midline glioma h3 k27-altered" OR AB="diffuse midline glioma h3 k27-altered") OR (TI="diffuse hemispheric glioma h3 g34-mutant" OR AB="diffuse hemispheric glioma h3 g34-mutant") OR (TI="infant-type hemispheric glioma" OR AB="infant-type hemispheric glioma") OR (TI=ganglioglioma OR AB=ganglioglioma) OR (TI="desmoplastic infantile astrocytoma" OR AB="desmoplastic infantile astrocytoma") OR (TI="desmoplastic infantile ganglioglioma" OR AB="desmoplastic infantile ganglioglioma") OR (TI="dysembryoplastic neuroepithelial tumour" OR AB="dysembryoplastic neuroepithelial tumour") OR (TI=dnet OR AB=dnet) OR (TI="multinodular vacuolating neuronal tumour" OR AB="multinodular vacuolating neuronal tumour") OR (TI=mvnt OR AB=mvnt) OR (TI="rosette-forming glioneuronal tumour" OR AB="rosette-forming glioneuronal tumour") OR (TI="papillary glioneuronal tumour" OR AB="papillary glioneuronal tumour") OR (TI="diffuse leptomeningeal glioneuronal tumour" OR AB="diffuse leptomeningeal glioneuronal tumour") OR (TI="myxoid glioneuronal tumour" OR AB="myxoid glioneuronal tumour") OR (TI=gangliocytoma OR AB=gangliocytoma) OR (TI="lhermitte-duclos disease" OR AB="lhermitte-duclos disease") OR (TI="central neurocytoma" OR AB="central neurocytoma") OR (TI="extraventricular neurocytoma" OR AB="extraventricular neurocytoma") OR (TI="cerebellar liponeurocytoma" OR AB="cerebellar liponeurocytoma") OR (TI=ependymoma OR AB=ependymoma) OR (TI="ependymoma zfta fusion-positive" OR AB="ependymoma zfta fusion-positive") OR (TI="myxopapillary ependymoma" OR AB="myxopapillary ependymoma") OR (TI=subependymoma OR AB=subependymoma) OR ALL=glioma) AND (((TI=procarbazine OR AB=procarbazine) AND (TI=lomustine OR AB=lomustine) AND (TI=vincristine OR AB=vincristine)) OR ((TI=pcv OR AB=pcv) AND (TI=pc OR AB=pc)))

**Table S2.** Treatment regimen description.

Study	Treatment details
Ahn *et al* [13]	After maximal safe resection, adjuvant therapy was tailored by glioma subtype: glioblastoma received standard chemoradiation with TMZ followed by six adjuvant cycles; grade II gliomas received radiotherapy alone, while grade III also included chemotherapy (PC or PCV). At recurrence, TMZ was preferred, with bevacizumab or nitrosourea-based regimens (PC or PCV) as alternatives. PC combined lomustine and procarbazine every 4 weeks, whereas PCV added vincristine and was given every 6 weeks.
Webre *et al* [11]	All patients underwent surgery, with extent of resection classified by postoperative imaging and records. All received PCV or PC chemotherapy, administered as primary therapy or after first or second progression.
Vesper *et al* [10]	Patients with WHO grade III oligodendrogliomas or oligoastrocytomas generally received postoperative radiotherapy (60 Gy in 30 fractions) plus chemotherapy with either PCV every 6 weeks or PC every 12 weeks, typically for ≥3 cycles and monitored by MRI/CT every 3 months. Treatment was discontinued after sustained response/stability or adjusted for toxicity. Selected small tumours were treated with I125 brachytherapy and microsurgical resection was reserved for accessible or refractory recurrences.
